# Brain potentials reveal reduced attention and error-processing during a monetary Go/No-Go task in procrastination

**DOI:** 10.1038/s41598-020-75311-2

**Published:** 2020-11-12

**Authors:** Jarosław M. Michałowski, Ewa Wiwatowska, Mathias Weymar

**Affiliations:** 1 Poznan Laboratory of Affective Neuroscience, Department of Psychology and Law, SWPS University of Social Sciences and Humanities, Kutrzeby 10 St., 61-719 Poznan, Poland; 2 Department of Biological Psychology and Affective Science, Faculty of Human Sciences, University of Potsdam, Potsdam, Germany; 3 Faculty of Health Sciences Brandenburg, University of Potsdam, Potsdam, Germany

**Keywords:** Motivation, Reward, Neurophysiology, Psychology, Cognitive control, Neuroscience, Attention

## Abstract

Procrastination is a self-regulatory problem of voluntarily and destructively delaying intended and necessary or personally important tasks. Previous studies showed that procrastination is associated with executive dysfunctions that seem to be particularly strong in punishing contexts. In the present event-related potential (ERP) study a monetary version of the parametric Go/No-Go task was performed by high and low academic procrastinators to verify the influence of motivational context (reward vs. punishment expectation) and task difficulty (easy vs. hard) on procrastination-related executive dysfunctions. The results revealed increased post-error slowing along with reduced P300 and error-related negativity (ERN) amplitudes in high (vs. low) procrastination participants—effects that indicate impaired attention and error-related processing in this group. This pattern of results did not differ as a function of task difficulty and motivation condition. However, when the task got more difficult executive attention deficits became even more apparent at the behavioral level in high procrastinators, as indexed by increased reaction time variability. The findings substantiate prior preliminary evidence that procrastinators show difficulties in certain aspects of executive functioning (in attention and error processing) during execution of task-relevant behavior, which may be more apparent in highly demanding situations.

## Introduction

Self-control is an essential human characteristic that can be defined as an ability to resist short-term temptations in order to achieve long-term goals^1^. It enables to maintain a good life quality in different domains, such as mental health, relationships quality or self-esteem^2^. Low self-control is associated with increased impulsivity and deficits in executive functions^3^, i.e. various cognitive processes that enable to adjust attention and behavior to changing internal states and external circumstances^4,5^. Self-control is also impaired in different subclinical and clinical conditions, such as neurodegenerative diseases^6,7^, affective disorders^8,9^ or attention deficit hyperactivity disorder^10^ (ADHD).

A psychological construct that has gained increased attention in recent years and has been linked to deficits in self-control is procrastination^11,12^. Procrastination is a phenomenon in which people voluntarily delay intended and necessary and/or personally important tasks, despite knowing that delaying will have more negative than positive consequences^13^. Procrastination affects 15–20% of the general population and even 80–95% of students^12^. The prevalence difference between general and student populations suggests that procrastination may be related to some situational factors. For instance, procrastinating increases with enhanced fatigue^14^ and insufficient sleep^15^; for tasks that are more difficult or boring; in contexts in which the risk of being evaluated increases; and when the moment of reward/punishment is distant in time^12,16^. Many of these conditions are met in the academic settings where voluntarily delaying of important study-related projects occurs despite expecting negative consequences, thus labeled as academic procrastination^12^. Our recent study also revealed that procrastinating in the academic setting escalated during COVID-19 lockdown due to additional situational factors, such as transition to on-line education or increased number of surrounding distractors^17^. Apart from the situational conditions, procrastination also depends on dispositional factors. A recent meta-analysis by Steel^12^ showed that procrastination shows a high degree of stability over time and is associated with other human characteristics that are considered to be stable traits, such as increased impulsiveness (r = 0.41) and decreased self-control (r = 0.58). Further studies also showed that procrastination is related to deficits in inhibition and goal management^18^ as well as to lower resistance to proactive interference^19^ and dysfunctions of executive attention^20–22^. Behavioral genetic studies confirmed that goal-management failures and impulsivity components such as tendencies to give into cravings and to act without thinking or planning strongly correlate with procrastination at the genetic and phenotypic level^18,23^. Procrastination is therefore also attributed to genetic influences however, reliable evidence on gene candidates linked to procrastination is still lacking.

Our own findings from two monetary Go/No-Go studies confirmed that procrastinators are characterized by increased impulsivity and executive dysfunctions^24,25^. In these studies, participants completed the Go/No-Go task in the standard version where subjects’ gratification did not rely on task performance. In addition, in these studies the Go/No-Go task was performed under different motivational conditions: under punishment condition where subjects’ errors resulted in losing some money that they received just before they started and under reward condition where subjects were paid for correct responses. In the first study^24^ the collected behavioral data revealed that the high (vs. low) procrastination group showed reduced post-error slowing (PES). The phenomenon of PES was first reported by Rabbitt^26^ as a typical slowdown in reaction time (RT) in trials following an error. Most conceptualizations of PES suggest that this is a cognitive adaptation mechanism of increased caution in responding aimed at reducing further errors^27,28^. In our study procrastinators did not increase their response caution after making an error, which is an evidence of error processing deficits in this group. This deficit was, however, only observed in punishment condition but not in reward and standard conditions suggesting that procrastinators’ executive deficits are particularly strong when punishment is expected. The second study^25^ confirmed the impairment in executive control in procrastinators in the punishment condition. In this functional brain imaging (fMRI) study, we found reduced activations in procrastinators during the whole punishment condition in anterior cingulate cortex (ACC) and right dorsolateral prefrontal cortex (dlPFC), regions that are related to self-control^29^. This finding confirmed our previous behavioral data showing that monetary punishment leads to lower executive control in procrastinators. Thus, albeit procrastinators did not show performance deficits in the number of errors in these tasks, the pattern of results suggests that procrastinators are prone to selective executive dysfunctions during expected punishment, as indexed by deficits in post-error slowing and reduced activity of ACC and dlPFC.

The present study aimed to continue this line of research and to replicate the findings of error-processing deficits in procrastination^24,25^ by using event-related potentials (ERPs). ERPs, compared to other neuroscientific measures (such as BOLD in fMRI) are highly suitable to track specific cognitive processes due to their high temporal resolution. This temporal sensitivity allows to identify the time course of error and stimulus processing in procrastination and to determine the moment at which specific impulsivity-related cognitive deficits become apparent (see below). For the present study, we focused on the error-related negativity (ERN)^30–33^, which is an ERP component that reliably occurs exactly at the moment when participants make an error, a component that has been considered to reflect an initial automatic error detection^31^. Prior research have found that the ERN is reduced in people with problematic impulsive behaviors such as substance use or behavioral addictions (for review see Luijten et al.^34^), indicating impaired error processing in these groups. Considering this and our own findings of procrastination-related error-processing deficits we expected to find decreased ERN in high (vs. low) procrastinators in the present study, particularly when the motivation is to avoid monetary punishment^24,25^.

The present study also aimed to expand our line of research in several directions. First, we intended to examine executive attention using the P300 component and the reaction time variability (RTV)^35–37^. Both variables correlate with each other^38^ and have been used as indicators of executive attention deficits, failures of inhibition and fluctuations in the efficiency of cognitive control^39–41^ in numerous previous studies, mainly in ADHD^42^. Taking into account previous findings showing executive attention deficits in procrastinators, we expected to find lower P300 amplitudes and higher reaction time variability (RTV) in this group. Secondly, because increasing task demands aggravate executive dysfunctions in groups characterized by self-control deficits^43–47^, a parametric Go/No-Go task (PGNG)^45^ was used in the present study. In this paradigm the Go/No-Go task is performed at two difficulty levels. On the first level, participants perform a usual Go/No-Go task, whereas on the second level they must simultaneously perform Go/No-Go and n-back tasks, which increases the working memory load. Based on previous findings of the relationship between task demands and executive deficits in high impulsive groups^16,24,25,48,49^, we supposed that the error processing and executive attention deficits would be more pronounced and would strongly influence behavior when the task is more difficult.

## Method

### Participants

179 students (48 male) from the University of Postdam completed the German version of the Aitken Procrastination Scale (APS-d)^50^. Based on the standard deviation of the mean in the central procrastination subscale of APS-d, the sample was divided into high (APS-d > 2.80; HP) and low (APS-d < 1.27; LP) procrastinators. Out of this sample, we have randomly chosen 25 students from each group (21 and 19 females in LP and HP, respectively). This group size was considered as sufficient based on a sample size estimation (α = 0.05; β = 0.20), which was performed based on data showing significant group differences in the ERN component between subjects with obsessive–compulsive disorder and controls^33^. We excluded participants with psychiatric or neurological illness as well as uncorrected vision. The study was approved by the local Ethics Committee at the University of Potsdam and performed in accordance with the Declaration of Helsinki. All participants signed informed consent prior participation, for which they, after completion, got credit points or 10 Euro (plus additional money they received in monetary Go/No-Go task, which was maximum 11 Euro).

### Measures

Academic procrastination was assessed with APS-d, which consists of three subscales: central procrastination, lack of foresight and unpunctuality^50^. The response format is a 5-point Likert scale with answers ranging from 0 (“not at all”) to 4 (“exactly right”). Participants also completed the German version of the UPPS Impulsive Behavior Scale, which measures 4 aspects of impulsivity: urgency, lack of premeditation, lack of perseverance and sensation seeking^51^. Answers are marked on a 4-point Likert scale ranging from 1 (“strongly agree”) to 4 (“strongly disagree”).

### Task and procedure

**Figure 1:**
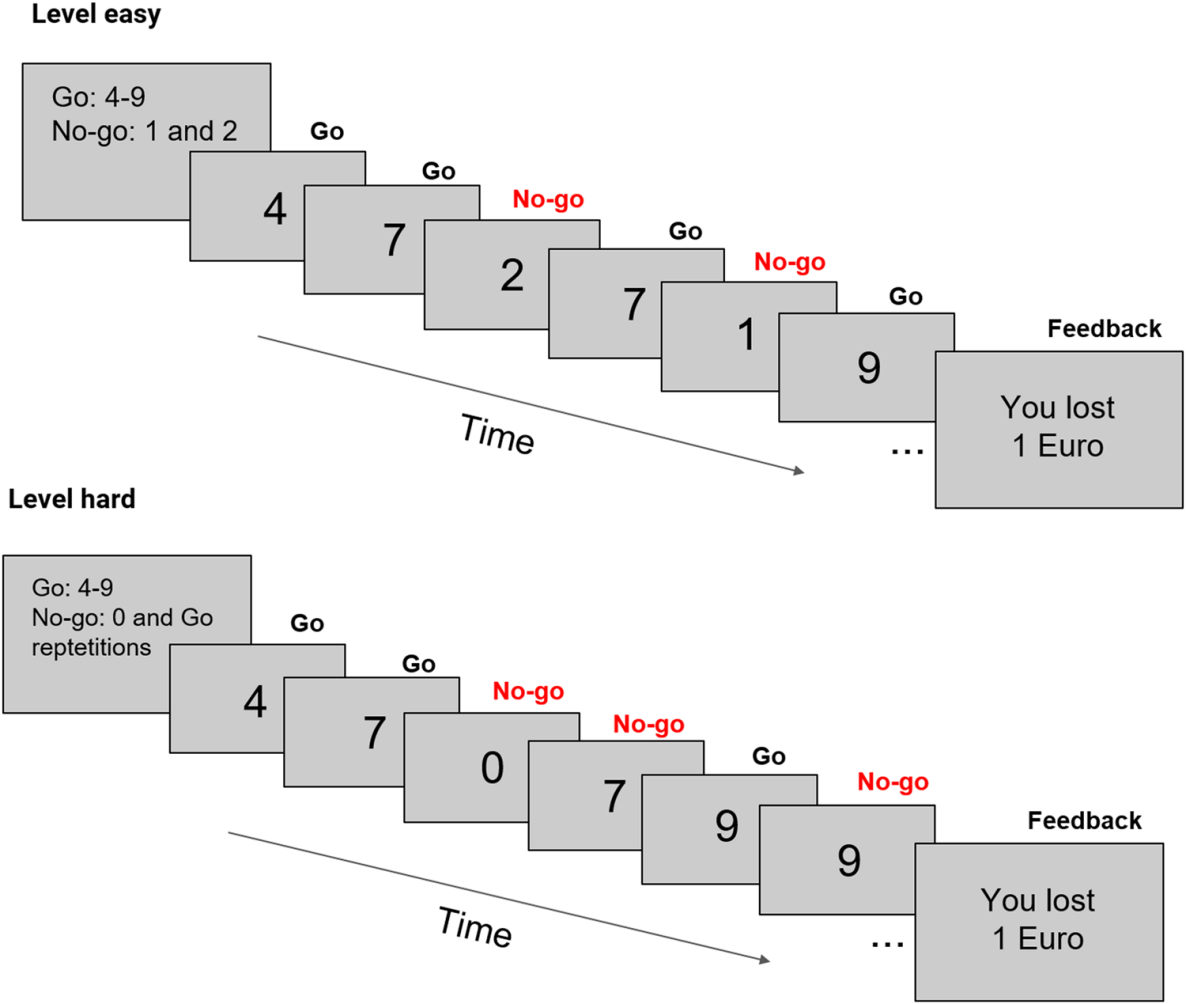
Parametric Go/No-Go Task. Each level consisted of 350 stimuli (5 blocks; 20% No-Go signals). On the first level of difficulty (top), digits 4–9 were used as Go and two other digits (here 1 and 2) as No-Go signals. On the second level (bottom), subjects had to withhold the reaction to one No-Go digit (here 0) and to repeated Go stimuli. Feedback presented at the end of each block indicated total loss (punishment condition) or profit (reward condition) for each block.

Participants completed a modified version of the Parametric Go/No-Go task (PGNG)^29^. During this task (see Fig. 1), digits that served as Go and No-Go signals, were presented for 250 ms on a monitor located in about 150 cm distance from participants’ eyes. Each digit was presented in one of five different fonts as in Michalowski et al.^24^. The inter-trial interval (ITI) was randomized between 1 (62.5% of ITIs), 2 (25%) and 3 s (12.5%). The PGNG task included two levels of difficulty, each comprised 5 blocks of 70 trials (20% of No-Go signals). On the first level (Fig. 1, top) participants had to push a response pad (with dominant hand) as fast as possible every time a Go stimulus (digits from 4 to 9) appeared on the screen and to withhold their reactions to No-Go stimuli (digits from 0 to 3). There were only two types of No-Go signals used at once and they changed every block. On the second level of difficulty subjects were instructed to inhibit their responses not only to No-Go stimuli (only one among 0–3 digits per block), but also to repeated Go stimuli (Fig. 1, bottom). The No-Go and the repeated Go stimuli occurred with a probability of 10% each. Each participant completed PGNG task in two conditions: monetary reward (REW) and punishment (PUN). In the REW condition participants gained 0.04 EUR for correct inhibitions and 0.01 EUR for reactions to Go signals that were faster than 500 ms with maximum possible gain of 11 Euro. In the PUN condition subjects were given 11 EUR prior to the experiment and each error resulted in a loss of small amount of money (0.04 and 0.01 EUR for false alarms to No-Go signals and missed/slow responses to Go signals, respectively). The order of conditions was counterbalanced across participants in both groups. The monetary and punishment condition lasted approximately 25 min each. Before the main part of the task, students completed a short training session (about 5 min). Presentation software (Neurobehavioral Systems, Inc., Berkeley, CA, https://www.neurobs.com) was used for stimulus presentation and recording of behavioral responses.

### Electrophysiological recordings and signal processing

Brain activity was continuously recorded from 129-channel HydroCel sensor nets using an Electrical Geodesics system (EGI Inc., Eugene, OR, USA) and digitized at a rate of 250 Hz, using the vertex sensor (Cz) as recording reference. The impedance of each sensor was kept below 50 kΩ or, in the case of the reference channel, below 5 kΩ, as recommended by the manufacture’s guidelines. Online, all channels were bandpass filtered (0.1 to 200 Hz). Offline, EEG signal processing was conducted with Electro Magneto Encephalography Software (v. 2.5; EMEGS)^52^. First, the data was segmented into epochs 400 ms before and 1000 ms after the stimulus or response and filtered with 0.1 Hz high-pass and 40 Hz low-pass filters. Then, eye movements were extracted with BioSig toolbox^53^ for MATLAB (v. R2013a, MathWorks) and artifact detection and correction was conducted according to the method described by Junghöfer, Elbert, Tuckert and Rockstroh^54^, which included conversion to average reference, interpolation of noisy channels and detection of artifactual segments. Mean number of interpolated electrodes was equal to 2.47 (*SD* = 1.94). Mean numbers of trials averaged in each condition and difficulty level for both groups are available in the supplementary materials. Data with a high number of artifactual epochs that exceeded the threshold of 25% were excluded from further analyses, which resulted in the exclusion of one subject from HP and four subjects from LP group. The file of one other participant from HP group was not usable due to hardware malfunction. The final sample therefore included 44 participants (21 and 23 in LP and HP, respectively).

**Figure 2:**
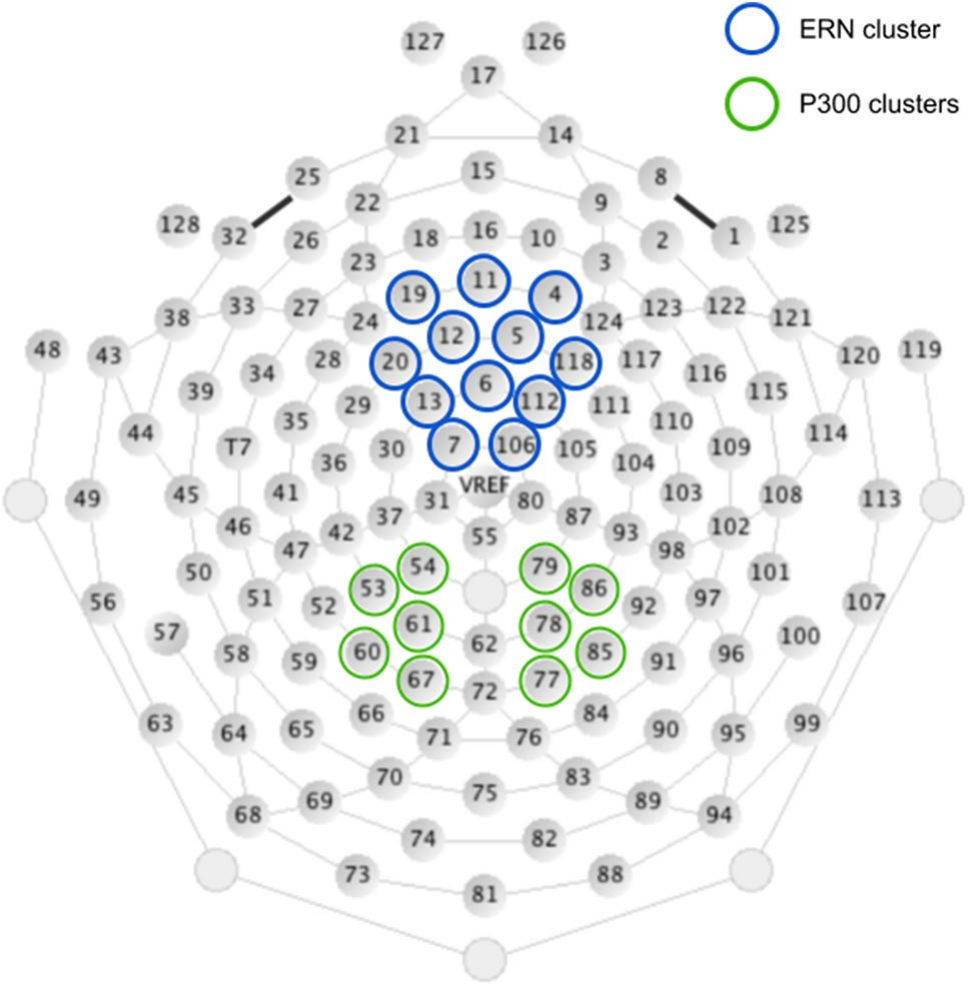
Sensor outline of the EGI HCGSN 130 electrode net (original image downloaded from https://www.egi.com/images/stories/manuals/Second%20Batch%20of%20IFUs%20with%20new%20Notified%20Body%20Jan%202019/GSN_tman_8105171-51_20181231.pdf). Sensors clusters used for ERP analysis are marked in blue (for ERN) and green (for P300).

Selection of time windows and channels for ERPs analyses was based on visual inspection of grand averages from both conditions in all participants and were consistent with prior research^55,56^. ERN was averaged within the time window from 34 ms before to 84 ms after committing a false alarm at frontal electrodes cluster (channels: 4, 5, 6, 7, 11, 12, 13, 19, 20, 106, 112, 118). P300 was averaged from the time window 260–380 ms after the stimulus onset at two parietal clusters (left: 53, 54, 60, 61, 67; and right: 77, 78, 79, 85, 86; see Fig. 2). As the P300 was delayed and located centrally for No-Go stimuli, additional analyses were performed for a central cluster (channels: 7, 31, 55, 80, 106, 129) and a 400–500 ms time window. The results of these analyses are available in the supplementary materials.

### Statistical analysis

Statistical analyses were conducted with IBM SPSS Statistics 25. Response times (RTs) were measured for correct responses only. Coefficient of variation (CV) was used as an indicator of reaction time variability (RTV), which was computed by dividing the standard deviation of RT by mean RT for each participant individually^57^. Post-error slowing (PES) was calculated as the difference between the mean RT to Go signals after false alarms and mean RT. Response accuracy was measured as the percentage of correct reactions for Go and No-Go trials separately.

Independent sample t-tests were carried out to compare group differences in the scores of the UPPS scales. Statistical analyses of behavioral (RT, Go and No-Go accuracy, PES, RTV) and ERN effects were conducted using repeated measures ANOVAs with condition (PUN vs. REW) and difficulty level (easy vs. hard) as within-group factors and procrastination (LP vs. HP) as between-subjects variable. For P300, the within factors stimulus (Go vs. No-Go) and laterality (right vs. left) were also included. Observations that exceeded the threshold of three standard deviations of the group’s mean were excluded. Thus, P300, RT, RTV and No-Go accuracy data from one subject as well as PES and Go accuracy data from two subjects were not included in the final analysis.

## Results

### Behavioral and questionnaire data

All behavioral results are presented in Table 1. Overall, participants responded faster (*F*(1,41) = 177.96; *p* < 0.001; *η*_*p*_^2^ = 0.81) and more accurate (*F*(1,40) = 8,86; *p* = 0.005; *η*_*p*_^2^ = 0.18 for Go; *F*(1,41) = 63.56; *p* < 0.001; *η*_*p*_^2^ = 0.61 for No-Go) during the low (vs. high) difficulty conditions. Go accuracy was higher among LP than HP (*F*(1,40) = 6.46; *p* = 0.015; *η*_*p*_^2^ = 0.14). No other main effects of group as well as no significant group × condition or group × difficulty interactions were observed for RTs or response accuracy (*Fs* < 1, *ps* > 1*)*.

**Table 1:** Means (SDs) of reaction times (RT), response accuracy, post-error slowing (PES) and reaction-time variability (RTV) for high (HP) and low (LP) procrastinators in the punishment (PUN) and reward (REW) condition.

	Condition	HP		LP	
		Level easy	Level hard	Level easy	Level hard
RT [ms]	PUN	346.90 (29.27)	391.30 (46.88)	350.29 (34.21)	400.28 (52.55)
	REW	346.76 (38.53)	394.80 (48.27)	354.40 (34.94)	396.95 (50.29)
No-Go accuracy [%]	PUN	78.90 (12.83)	67.60 (14.28)	76.67 (14.92)	68.98 (12.53)
	REW	74.48 (15.02)	66.56 (17.06)	76.67 (13.38)	69.05 (15.06)
Go accuracy [%]	PUN	99.63 (0.74)	99.50 (0.51)	99.91 (0.20)	99.70 (0.46)
	REW	99.61 (0.62)	99.25 (0.88)	99.91 (0.20)	99.77 (0.35)
PES [ms]	PUN	0.80 (35.50)	17.91 (36.30)	− 7.06 (22.14)	− 3.41 (31.70)
	REW	5.83 (28.54)	25.61 (31.43)	− 7.47 (23.69)	9.03 (38.04)
RTV	PUN	0.19 (0.04)	0.22 (0.04)	0.18 (0.04)	0.20 (0.03)
	REW	0.19 (0.04)	0.24 (0.05)	0.19 (0.04)	0.21 (0.03)

**Figure 3:**
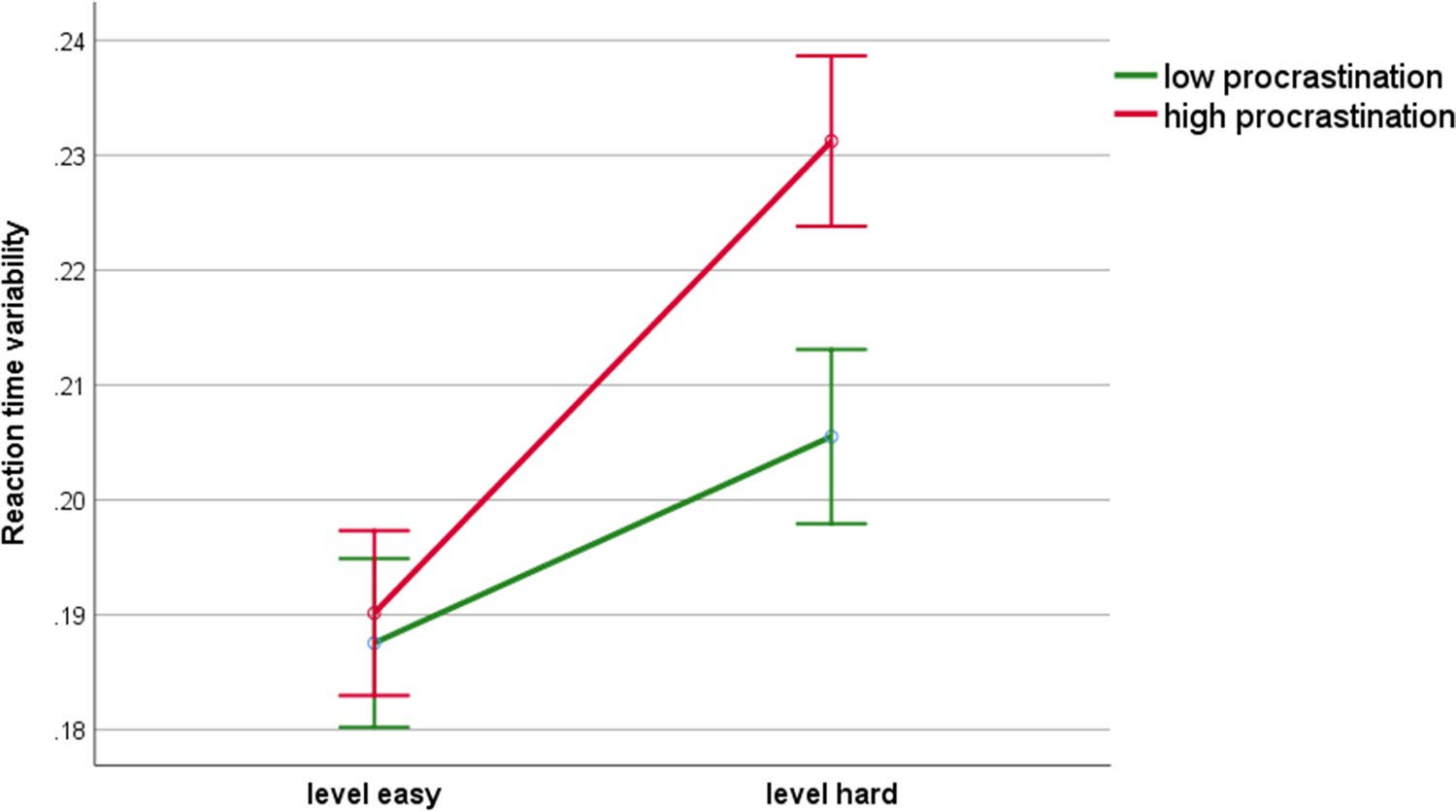
Reaction time variability in high and low procrastinators as a function of difficulty level in the PGNG task. Error bars represent one standard error.

**Figure 4:**
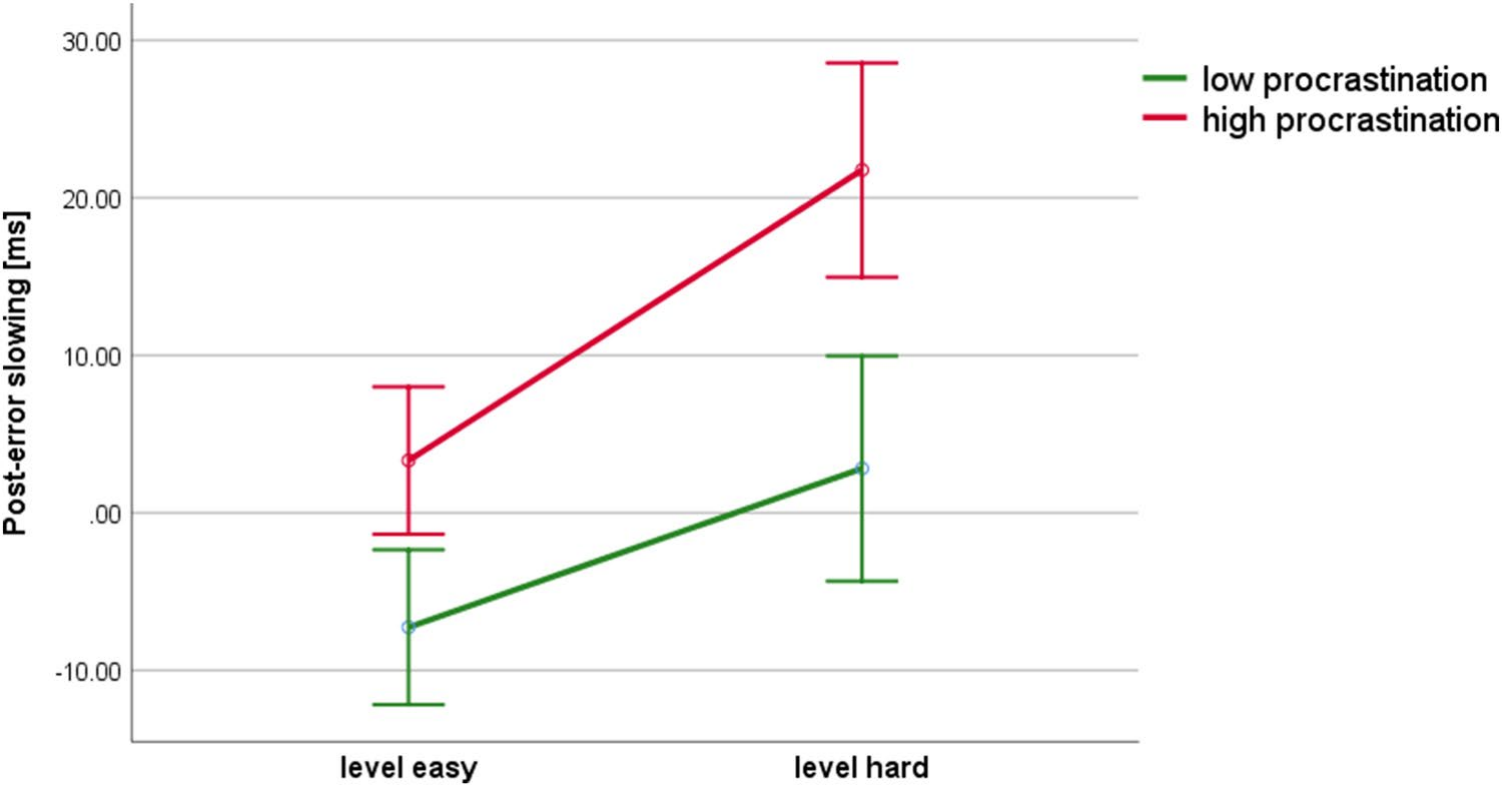
Differences in post-error slowing between high and low procrastinators. Error bars represent one standard error.

PES and RTV were increased during reward (REW) compared to punishment (PUN) condition (*F*(1,38) = 4.24; *p* = 0.047; *η*_*p*_^2^ = 0.10 for PES; *F*(1,41) = 6.14; *p* = 0.017; *η*_*p*_^2^ = 0.13 for RTV) and in high (vs. low) difficulty condition (*F*(1,38) = 7.61; *p* = 0.009; *η*_*p*_^2^ = 0.17 for PES; *F*(1,41) = 56.78; *p* < 0.001; *η*_*p*_^2^ = 0.58 for RTV). Furthermore, RTV was higher in high (vs. low) procrastinators, but only in high difficulty condition (Group × Difficulty: *F*(1,41) = 8.70; *p* = 0.005; *η*_*p*_^2^ = 0.18; see Fig. 3). PES was more pronounced in HP than LP on both difficulty levels (*F*(1,38) = 4.85; *p* = 0.034; *η*_*p*_^2^ = 0.11; see Fig. 4). The group effects were similar regardless of motivation condition for both PES and RTV (Group × Motivation and Group × Motivation × Difficulty: *F*s < 1, *p*s > 0.1).

Independent sample t-tests showed that HP participants scored higher than LP students in two out of four UPPS subscales: Urgency (*M*_LP_ = 25.35, *SD*_LP_ = 5.03; *M*_HP_ = 29.74, *SD*_HP _= 6.89; *t*(41) = 2.35, *p* = 0.023; *d* = 0,73) and Lack of perseverance (*M*_LP_ = 16.55, *SD*_LP_ = 3.46; *M*_HP_ = 22.61, *SD*_HP_ = 4.63; *t*(41) = 4.80, *p* < 0.001; *d* = 1.48).

### Event-related potentials

**Figure 5:**
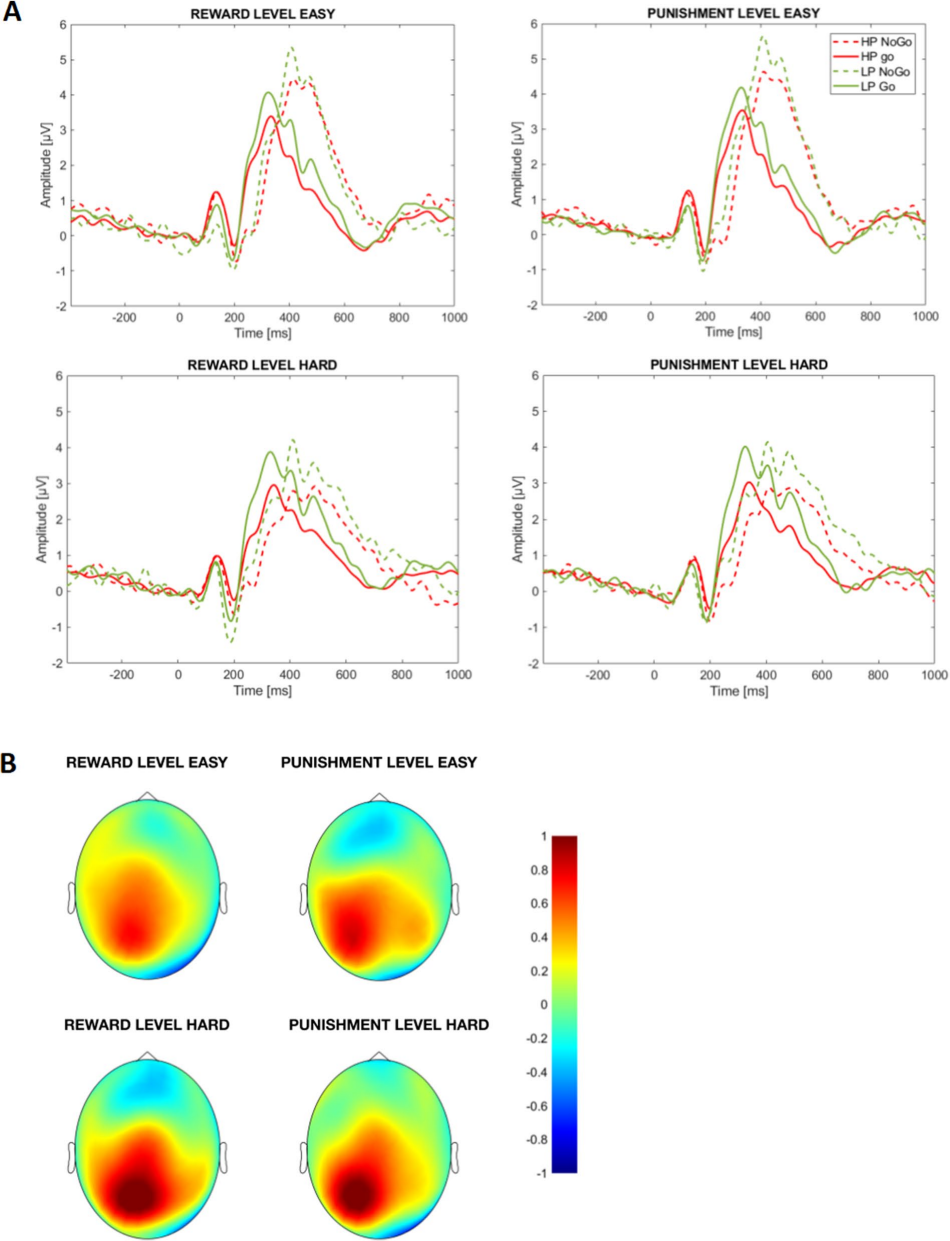
(**A**) Stimulus-locked ERPs averaged over left parietal cluster displayed for Go and No-Go stimuli in the high (HP) and low (LP) procrastination group; (**B**) Scalp potential difference between HP and LP in 260–380 time window after stimulus averaged for Go and No-Go signals.

**Figure 6:**
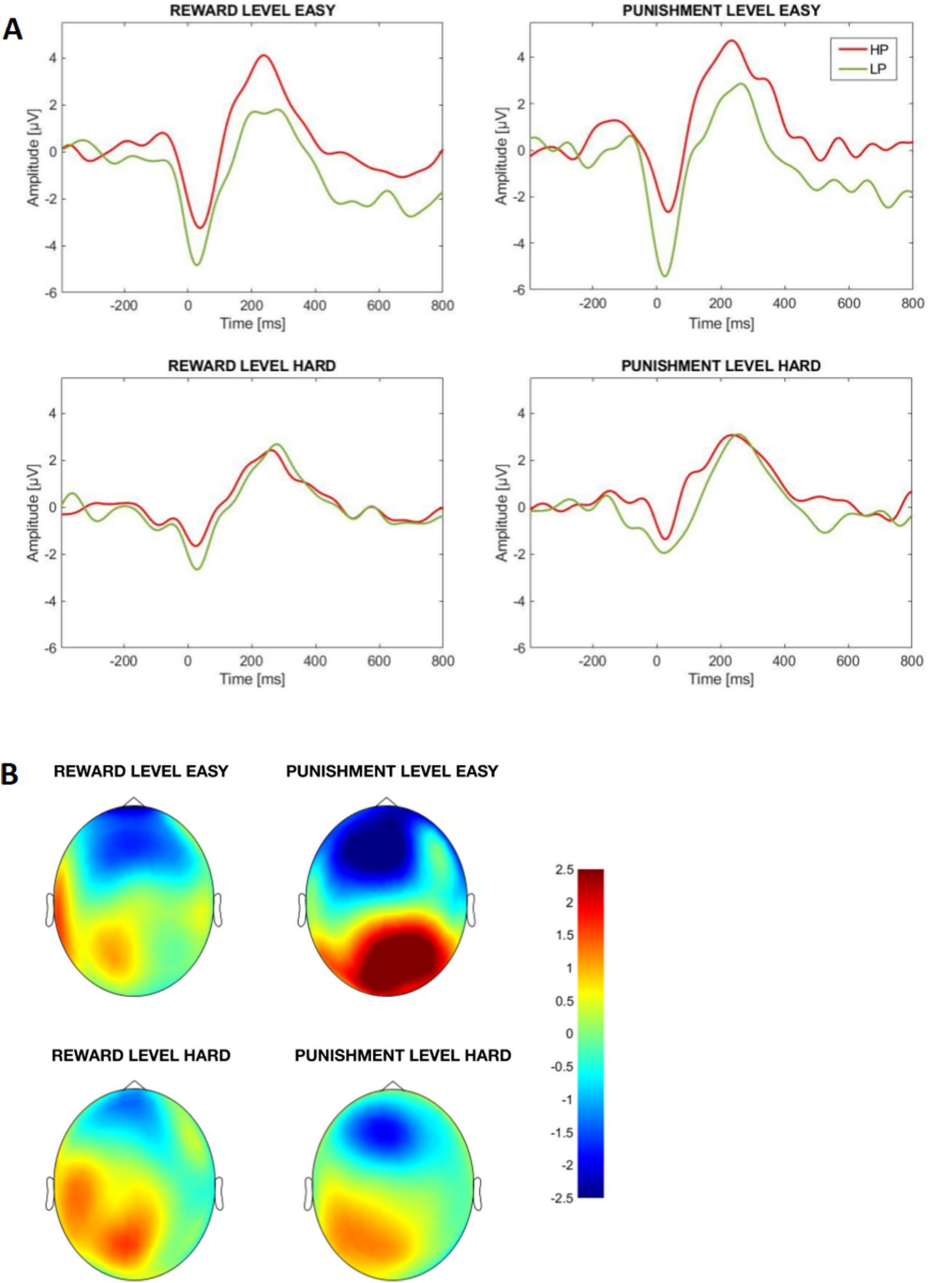
(**A**) ERPs elicited over frontal cluster by responses to No-Go signals in high (HP) and low (LP) procrastinators; (**B**) Scalp potential difference between HP and LP in a time window from 34 before to 84 ms after responses to No-Go signals.

Analyses of the P300 revealed that, overall, lower P300 amplitudes were observed during high (vs. low) difficulty condition, irrespective of motivation condition (*F*(1,41) = 28.67; *p* < 0.001; *η*_*p*_^2^ = 0.41). Further, HP responded with lower P300 amplitudes than LP over the left (but not right) hemisphere (interaction Group × Laterality: *F*(1,41) = 4.18; *p* = 0.047; *η*_*p*_^2^ = 0.09; see Fig. 5). No significant Group × Difficulty or Group × Condition interactions were observed (*Fs* < 1, *ps* > 1). Analyses of the ERN showed less pronounced negativity to errors in low (vs. high) difficulty levels for both groups and motivation conditions (*F*(1,35) = 20.09, *p* < 0.001; *η*_*p*_^2^ = 0.37). We also observed less pronounced ERN in HP than LP group (*F*(1,35) = 4.07, *p* = 0.052; *η*_*p*_^2^ = 0.10; see Fig. 6) regardless of motivation condition or difficulty level (*Fs* < 1, *ps* > 1 for both Group × Difficulty and Group × Motivation interactions).

## Discussion

The present study aimed at investigating behavioral and neuronal correlates of putative executive dysfunctions associated with procrastination. Furthermore, we were interested whether these executive dysfunctions differ as a function of task difficulty and type of motivation (reward vs. punishment). To achieve these goals a monetary version of the parametric Go/No-Go task was performed by low and high academic procrastinators. The results showed lower Go accuracy and increased PES along with reduced P300 and ERN in high, compared to low academic procrastination group, indicating impaired attention-and error-related processing. This pattern of results did not differ as a function of task difficulty and motivation condition. However, when the task got more difficult reaction time began to fluctuate to a greater extent in high when compared to low procrastinators.

The present ERP results revealed that procrastination is associated with reduced error-related neuronal activity, as indexed by lower ERN amplitudes. ERN is proposed to reflect initial automatic error detection and was shown to originate from the ACC that integrates cognitive and affective processes during error detection^58–61^. Interestingly, ACC activity is also reduced after errors in procrastinators as demonstrated by Wypych and collaborators^25^, which is in line with the present ERP findings. ACC activity and ERN amplitudes are commonly observed to be reduced in problematic impulsive behaviors related to externalizing disorders, such as substance use disorders and ADHD^34,62^. Reduced ERN is assumed to result from low dopaminergic input that usually convey a negative enforcement signal to activate ACC neurons in order to modify behavior after an error^63^. Pharmacological studies indeed confirmed that decreased ERN amplitudes are related to lower dopaminergic activity^64,65^. Further, the ERN amplitude was shown to decrease when the goals that could have been achieved during the task were evaluated as less valuable or significant by the subject^59^. This suggests that the error-processing activity depends not only on dispositional factors but also on motivation to engage in a goal achievement activity (for the illustration of the interaction between motivation and procrastination see Temporal Motivation Theory formulated by Steel and Koenig^66^). Based on that we conclude that the reduced ERN amplitudes in academic procrastinators may be related to problems in allocating resources for error processing and/or reduced motivational engagement.

The present attention-related ERP results are also in line with the above conclusions. We found generally reduced P300 amplitudes in procrastinators indicating that the task engages less attentional resources in this group. This is supported by the behavioral data in which a larger number of omission errors were found in high procrastination group. Importantly, when the task became more difficult, procrastinators showed higher intraindividual reaction time variability, an effect suggesting fluctuations in executive control^40,41^ or attentional lapses^39^. In the context of a reduced P300 in procrastinators we propose that this behavioral effect expresses procrastinators’ deficits in attentional control. The ability to control attention requires to constantly monitor and inhibit other thoughts, feelings, and responses. This ability is also called executive attention and was shown to be linked to the activity of the ACC and the lateral prefrontal cortex (LPFC)^67,68^ as well as to impaired dopaminergic neurotransmission (linked to attentional and behavioral control deficits)^69–71^. Our findings are also consistent with other studies showing executive attention dysfunctions in procrastinators^12,72^.

Contrary to our expectations, though, procrastination-related decrease in brain responses related to executive control (i.e. reduced ERN and P300) was not affected by task difficulty. The between-group differences in ERPs revealed that the neuronal dysfunctions observed in procrastinators when the working memory load was high (Go/No-Go combined with n-back task) were similar to those observed during the simple Go/No-Go task. However, with increasing memory load these neuronal dysfunctions became more apparent at the behavioral level—the increase in difficulty was accompanied by higher reaction time variability in high (vs. low) procrastinators indicating behavioral executive attention deficits in this group. This is in line with previous findings that high difficulty level is more sensitive in detecting executive deficits in high-impulsivity populations^43–45,47^. At the same time, our study also shows that ERPs are sensitive in detecting executive deficits even at lower working memory load.

Interestingly, PES was increased in high compared to low academic procrastinators. This was opposite of what we expected and suggests that procrastinators apply more cognitive control after committing an error to decrease the possibility of another mistake. However, our interpretation here is different and based on the assumption that PES may also result from reallocation of resources after errors. With regard to previous studies showing that the immediate slowing after errors is associated with a reduction in sensory sensitivity and motor inhibition which may result from an orienting response to errors^73,74^, we suggest, again, that procrastinators have problems with executive control and that they need more time to reorient their attention from committed mistake to response execution in the following trial. Due to the inclusion of a high difficulty condition, the present experimental procedure may have been more challenging than in the previous studies^24,25^ and could have consumed more attentional resources, especially in high procrastinators.

The procrastination-related effects observed in the present study were not affected by the type of motivation that was induced in our paradigm. This is in contrast to our expectations and our previous fMRI findings which found reduced ACC and dLPFC activity in high vs. low procrastinators in the punishment but not in the reward condition^25^. The results of the present EEG and the recent fMRI study are, however, difficult to compare. First, due to differences in the timing of stimulus presentation, slightly different cognitive processes might have been engaged in the fMRI and in the present study. Second, the fMRI findings showed that expectation of monetary punishment resulted in tonic (but not error-related) reduction in ACC and dLPFC activity in procrastinators. This effect was interpreted as being indicative of procrastination-related deficits in proactive control rather than in error processing. Proactive control was not investigated in the current research, although the increased RTV observed in HP in the present study may support this interpretation^75^. Both EEG and fMRI results indicate that the use of financial punishment versus reward conditions is insufficient to differentially influence error and stimulus processing in procrastinators. This was confirmed by the present behavioral data since motivation condition did not influence PES and RT variability in procrastinators.

There are certain limitations that may have influenced the present findings. First, the relatively small sample size limited the statistical power and might have been responsible for the fact that the between group effects were weak. Second, the present findings have been obtained with the academic sample and, although the strong association between general and academic procrastination has been repeatedly reported^76^, they cannot be directly transferred to other populations without additional investigations. Third, future studies may consider investigating the influence of social evaluation rather than financial motivation, as this may be more appropriate for the sample that is characterized by increased fear of failure and negative evaluation^16,48,49^. And finally, using the advantages of EEG future studies may try to determine the relationship between procrastination and the temporal characteristics of the processing flow using various latency measures and techniques (e.g. steady-state visual evoked potentials, ssVEPs), as the present behavioral data indicate that high and low procrastinators may differ in the timing of the specific cognitive processes (e.g. due to attention fluctuation).

In summary, the present data confirmed previous findings of executive dysfunctions in academic procrastination. These dysfunctions can be observed at the neuronal and behavioral level even when the task is relatively easy, but they become more pronounced behaviorally when working memory load increases. A detailed look at the pattern of the present findings indicated that procrastinators have specific performance monitoring and executive attention deficits. This is similar to the pattern observed in individuals with ADHD that are characterized by reduced ERN^77^ and P300^78^ and increased RTV^79–81^. Executive and attentional deficits observed in ADHD were suggested to be related to reduced cortical arousal caused by decreased tonic activity of the noradrenergic system^82^. These deficits are reduced when ADHD is treated with Metylphenidad^83,84^, which again suggests that they are mediated by dysfunctions of noradrenergic and dopaminergic mechanisms. Procrastination problems have been observed in ADHD patients and are commonly treated in this group^72,85^. Our present and previous neuroimaging data^25^ suggests that procrastination develops partly due to similar neuronal dysfunctions as those observed in ADHD and other groups with self-control problems. However, the relation between procrastination and these disorders has not yet been clarified and therefore needs further research.

## Supplementary information

Supplementary Information.
