# Supplementary material for: Brain potentials reveal reduced attention and error-processing during a monetary Go/No-Go task in procrastination
Source: Sci Rep. 2020 Nov 12;10:19678. doi: 10.1038/s41598-020-75311-2 (PMC7661523; doi:10.1038/s41598-020-75311-2)
Supplement: Supplementary file 1 — Supplementary Information. [file 41598_2020_75311_MOESM1_ESM.pdf]

Supplementary materials for
Brain potentials reveal reduced attention and error-processing during a monetary Go/No-Go task in procrastination.

Jarosław M. Michałowski[1*], Ewa Wiwatowska[1] & Mathias Weymar,[2,3]

[1]Poznan Laboratory of Affective Neuroscience, Department of Psychology and Law SWPS University of Social Sciences and Humanities, Poznań, Poland;

[2]Department of Biological Psychology and Affective Science, Faculty of Human Sciences, University of Potsdam, Potsdam, Germany;

[3]Faculty of Health Sciences Brandenburg, University of Potsdam, Potsdam, Germany;

SUPPLEMENTARY METHODS

Table S1. Mean numbers (SDs) of trials averaged for high (HP) and low (LP) procrastinators for each condition and difficulty level.

		LP		HP	
Trial type	Difficulty level	Punishment	Reward	Punishment	Reward
Go	easy	248,81 (12,73)	252,30 (15,66)	243,14 (12,84)	239,95 (13,80)
	hard	228,29 (20,18)	222,95 (46,32)	225,91 (13,46)	229,50 (17,60)
No-Go	easy	50,62 (3,97)	58,80 (6,10)	57,23 (5,50)	55,14 (6,39)
	hard	55,62 (6,14)	53,05 (11,89)	53,27 (6,93)	54,05 (6,51)
False alarm	easy	14,06 (8,46)	13,00 (7,34)	11,35 (10,51)	13,35 (10,10)
	hard	15,29 (6,80)	14,75 (8,77)	14,15 (11,04)	16,50 (11,38)

SUPPLEMENTARY RESULTS

Table S2. Multiple measures ANOVA effects calculated for mean P300 amplitudes scored from two parietal clusters (left: 53, 54, 60, 61, 67; and right: 77, 78, 79, 85, 86) at the time window 260-380 ms after stimulus onset.

Effect	F value	p value	Partial eta-squared
stimulus	86,11	<.001	.677
stimulus x group	0,13	.725	.003
laterality	1,30	.260	.031
laterality x group	4,18	.047	.092
level	28,67	<.001	.411
level x group	0,52	.475	.012
condition	1,51	.226	.035
condition x group	0,10	.756	.002
stimulus x laterality	0,68	.413	.016
stimulus x laterality x group	0,01	.909	<.001
stimulus x level	0,48	.494	.011
stimulus x level x group	1,30	.261	.031
laterality x level	0,38	.541	.009
laterality x level x group	0,45	.507	.011
stimulus x laterality x level	0,27	.604	.007
stimulus x laterality x level x group	1,02	.319	.024
stimulus x condition	0,01	.906	<.001
stimulus x condition x group	0,25	.619	.006
laterality x condition	0,55	.463	.013
laterality x condition x group	1,08	.304	.026
stimulus x laterality x condition	0,74	.396	.018
stimulus x laterality x condition x group	4,11	.049	.091
level x condition	0,10	.749	.003

level x condition x group	1,26	.268	.030
stimulus x level x condition	0,05	.831	.001
stimulus x level x condition x group	0,57	.455	.014
laterality x level x condition	1,48	.231	.035
laterality x level x condition x group	1,07	.307	.025
stimulus x laterality x level x condition	0,28	.601	.007
stimulus x laterality x level x condition x group	0,05	.833	.001
group	2,63	.113	.060

Table S3. Multiple measures ANOVA effects calculated for mean ERN amplitudes scored from frontal electrodes cluster (channels: 4, 5, 6, 7, 11, 12, 13, 19, 20, 106, 112, 118) at the time window from 34 ms before to 84 ms after the false alarm.

Effect	F value	p value	Partial eta-squared
level	20,09	<.001	.365
level x group	0,34	.566	.009
condition	3,10	.087	.081
condition x group	0,42	.523	.012
level x condition	0,01	.916	<.001
level x condition x group	0,44	.514	.012
group	4,07	.052	.104

Table S4. Multiple measures ANOVA effects for mean P300 amplitudes scored from fronto-central electrodes cluster (channels 7, 31, 55, 80, 106, VREF) at the time window 400-500 after stimulus onset.

Effect	F value	p value	Partial eta-squared
stimulus	156,24	<.001	.792
stimulus x group	0,09	.762	.002
level	38,34	<.001	.483
level x group	0,19	.665	.005
condition	1,05	.312	.025
condition x group	0,44	.511	.011
stimulus x level	102,60	< .001	.714
stimulus x level x group	0,30	.586	.007
stimulus x condition	1,25	.271	.030
stimulus x condition x group	< 0,01	.991	< .001
level x condition	0,48	.493	.012
level x condition x group	0,01	.915	< .001
stimulus x level x condition	0,69	.411	.017
stimulus x level x condition x group	0,17	.680	.004
group	0,03	.852	.001
